# Conjugate heat transfer of laminar mixed convection of a nanofluid through an inclined tube with circumferentially non-uniform heating

**DOI:** 10.1186/1556-276X-6-360

**Published:** 2011-04-26

**Authors:** Shahriar Allahyari, Amin Behzadmehr, Seyed Masoud Hosseini Sarvari

**Affiliations:** 1 Mechanical Engineering Department, University of Sistan and Baluchestan, P.O. Box 98164-161, Zahedan, Iran; 2 Mechanical Engineering Department, Shahid Bahonar University, Kerman, Iran

## Abstract

Laminar mixed convection of a nanofluid consisting of water and Al_2_O_3 _in an inclined tube with heating at the top half surface of a copper tube has been studied numerically. The bottom half of the tube wall is assumed to be adiabatic (presenting a tube of a solar collector). Heat conduction mechanism through the tube wall is considered. Three-dimensional governing equations with using two-phase mixture model have been solved to investigate hydrodynamic and thermal behaviours of the nanofluid over wide range of nanoparticle volume fractions. For a given nanoparticle mean diameter the effects of nanoparticle volume fractions on the hydrodynamics and thermal parameters are presented and discussed at different Richardson numbers and different tube inclinations. Significant augmentation on the heat transfer coefficient as well as on the wall shear stress is seen.

## Introduction

Many different industries such as electronic, automotive and aerospace have been facing heat transfer limitation for improving performance of their thermal systems. Heat transfer enhancement has been considered as one of the key parameter for developing more efficient and effective thermal devices. Thus this issue has been studied extensively. Different active and passive methods have been considered for the heat transfer augmentation. Improving the thermo-physical properties of the working fluids such as water, oil and ethylene glycol mixture is one of the possible methods. Therefore, there has been a strong motivation to develop a new heat transfer fluids with substantially higher thermal conductivity. Choi [1] presented a new generation of solid-liquid mixtures that is called nanofluid. It demonstrates significant improvement over the thermal characteristics of the base fluids. Various nanofluids with different nanoparticle and base fluid materials have been prepared and their thermo-fluid characteristics have been investigated by many researchers.

Among them, experimental studies of [2-5] on confined geometries could be cited. In general they found that the Nusselt number increases with the nanoparticle concentrations and significant heat transfer enhancement has been achieved. Many works have been dedicated to determine and model the effective physical properties of different nanofluid. For instance, investigations of Refs. [6-13] on the effective thermal conductivity or the works that have been done by [14,15] on the nanofluid effective viscosity could be mentioned.

Convective heat transfer with nanofluids can be modelled using the two-phase or single-phase approach. The first provides the possibility of understanding the function of both the fluid phase and the solid particles in the heat transfer mechanisms. The second assumes that the fluid phase and particle are in thermal and hydrodynamic equilibrium. This approach is simpler and requires less computational time. Thus it has been used in several theoretical studies of convective heat transfer with nanofluids [16-18]. However, the concerns in single-phase modelling consist in selecting the proper effective properties for nanofluids and taking into account the chaotic movement of ultra fine particle. To partially overcome this difficulty, some researches [19-21] used the dispersion model which takes into account the improvement of heat transfer due to the random movement of particles in the main flow. In addition several factors such as gravity, friction between the fluid and solid particles and Brownian forces, the phenomena of Brownian diffusion, sedimentation and dispersion may coexist in the main flow of a nanofluid. This means that the slip velocity between the fluid and particle may not be zero [22]. Therefore, it seems that the two-phase approach could better model nanofluid behaviours. Behzadmehr et al. [23] studied the turbulent forced convection of a nanofluid in a circular tube by using a two-phase approach. They implemented the two-phase mixture model for the first time to study nanofluid. Their comparison with the experimental results showed that the two-phase mixture model is more precise than the single-phase model. Mirmasoumi and Behzadmehr [24] studied the laminar mixed convection of a nanofluid in a horizontal tube using two-phase mixture model. They showed that the two-phase mixture model could better simulate the experimental results than the single-phase model. Recently, Lotfi et al. [25] studied two-phase Eulerian model that has been implemented to investigate such a flow field. Their comparison of calculated results with experimental values shows that the mixture model is more precise than the two-phase Eulerian model.

This work intends to investigate conjugate mixed convection-conduction of a nanofluid though an inclined tube. The tube is subjected to a uniform heat flux on its top surface; it is insulated on its bottom surface. Therefore, the effects of tube inclinations and particle volume fractions on the hydrodynamic and thermal parameters have been presented over a wide range of Re-Gr combinations.

## Mathematical formulation

**Figure 1:**
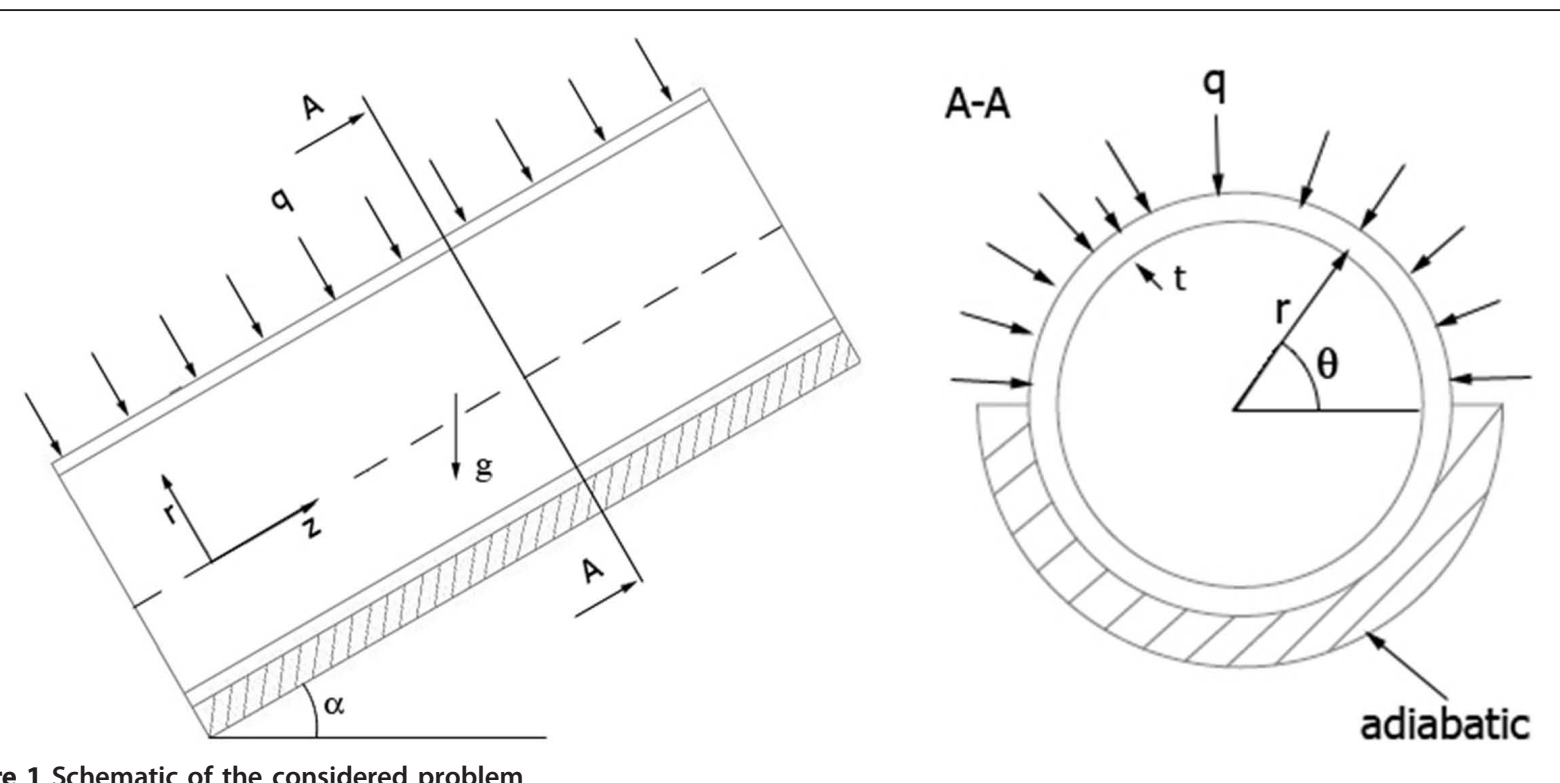
**Schematic of the considered problem**.

Mixed convection of a nanofluid consists of water and Al_2_O_3 _in a long copper tube with uniform heat flux at the top surface of tube wall has been considered. Figure 1 shows the geometry of the considered problem. The physical properties of the fluid are assumed constant except for the density in the body force, which varies linearly with the temperature (Boussinesq's hypothesis). Dissipation and pressure work are neglected. Thus, with these assumptions the conservation equations for steady state mixture model are as follows:

Continuity equation:(1)

Momentum equation:(2)

Energy equation for fluid:(3)

The heat conduction throughout the solid wall:(4)

Volume fraction:(5)

Where(6)

are the mean axial velocity and shear stress, respectively, and *ϕ *is the volume fraction of phase k.

In Equation 2, *V*_dr,k _is the drift velocity for the secondary phase k, i.e. the nanoparticles in the present study:(7)

The slip velocity (relative velocity) is defined as the velocity of a secondary phase (p) relative to the velocity of the primary phase (f):(8)

The drift velocity is related to the relative velocity:(9)

The relative velocity is determined from Equation 10 proposed by Manninen et al. [26] while Equation 11 by Schiller and Naumann [27] is used to calculate the drag coefficient:(10)(11)

The acceleration *a *in Equation 10 is:(12)

where:(13)

The physical properties in the above equations are:

Effective density:(14)

Chon et al. [12] correlation which considers the Brownian motion and nanoparticle mean diameter has been used for calculating the effective thermal conductivity:(15)

where *Pr *and *Re *in Equation 15 are defined as:(16)

*L*_f _= 0.17 nm is the mean free path of water, *B*_c _is the Boltzmann constant (1.3807 × 10^-23 ^J/K) and *μ *is calculated by the following equation:(17)

Thermal expansion coefficient Khanafer et al. [16]:(18)

An accurate equation is used for calculating the effective heat capacity [28].(19)

Effective viscosity is calculated by the following equation proposed by Masoumi et al. [15] which considers the effects of volume fraction, density and average diameters of nanoparticle and physical properties of the base fluid:(20)

## Boundary condition

This set of nonlinear elliptical governing equations has been solved subject to the following boundary conditions:

At the tube inlet (*Z *= 0):(21)

At (*r *= *r*_o_)(22)(23)

At the interface between the tube wall (copper) and the fluid (*r *= *r*_i_), the continuity condition for temperature and heat flux are applied so that:(24)

At the tube outlet: atmospheric static pressure is assumed.

## Numerical method and validation

**Figure 2:**
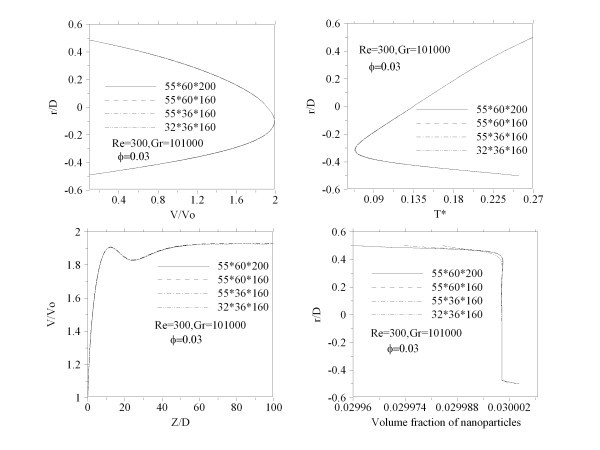
**Grid independence test**: **(a) **centerline axial velocity, **(b) **fully developed temperature, **(c) **fully developed velocity and **(d) **distribution of nanoparticles volume fraction.

This set of coupled non-linear differential equations was discretized with the finite volume technique. For the convective and diffusive terms a second order upwind method was used while the SIMPLEC procedure was introduced for the velocity-pressure coupling. The discretization grid is uniform in the circumferential direction and non-uniform in the other two directions. It is finer near the tube entrance and near the wall where the velocity and temperature gradients are important. Several different grid distributions have been tested to ensure that the calculated results are grid independent. The selected grid for the present calculations consists of 160, 32 and 36 nodes, respectively, in the axial, radial and circumferential directions. As shown in Figure 2 increasing the grid numbers does not significantly change the velocity and temperature of the nanofluid. The grid test on the nanoparticle volume fraction is shown in Figure 2d. It is seen that the nanoparticle concentration does not change with increasing the grid numbers in the radial direction. Other axial and radial profiles have also been verified to be sure the results are grid independent.

**Figure 3:**
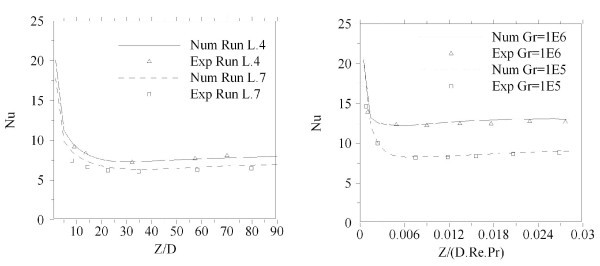
**Comparison of the axial evolution of Nu in a horizontal tube with the corresponding experimental results of (a) Barozzi et al. **[28]**, (b) Peutkhov et al. **[29].

In order to demonstrate the validity and also precision of the model and the numerical procedure, comparisons with the previously published experimental and numerical results have been done. Figure 3a,b shows the comparison of the calculated Nusselt number with the experimental results of Barozzi et al. [29] and Peutkhov et al. [30] in a horizontal tube, respectively. As shown good agreement between the results are seen.

**Figure 4:**
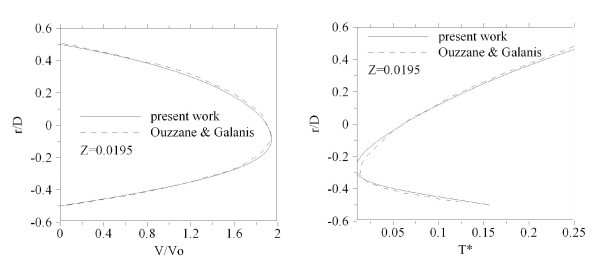
**Comparison of dimensionless velocity and temperature profiles in a horizontal tube with the corresponding numerical results **[30].

A comparison has also been performed with the numerical results obtained by Ouzzane and Galanis [31]. As shown in Figure 4, axial evolution of the dimensionless temperatures and velocity is in good concordance with the present results.

It should be mentioned that our numerical results were obtained using the two-phase mixture model and considering a very small volume fraction for the solid particles. Therefore, the numerical procedure is reliable and can well predict developing mixed convection flow in a tube.

## Results and discussions

Calculations have been performed over wide range of *Re*-*Gr *combinations and nanoparticle concentrations. The Grashof number (or Richardson number) has been limited in order to respect the validity of the Boussinesq's approximation for the fluid density variation. The results presented here are for different Richardson numbers and three nanoparticle volume fractions.

**Figure 5:**
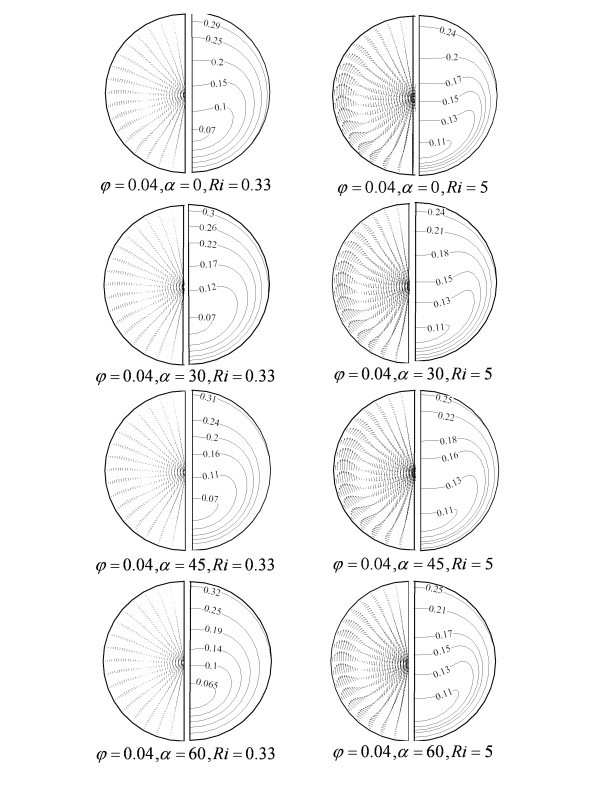
**Vectors of secondary flow and Contours of dimensionless temperature for different Ri and tube inclinations**.

For a given nanoparticle mean diameter and concentration (*d*_p _= 28 nm, *Φ *= 0.04) the effect of tube inclinations on the secondary flow vector and dimensionless temperature are shown in Figure 5 for two different Richardson numbers. As mentioned the tube is considered to be made of copper which is a high thermal conductive metal. This transfers the heating energy from the top half surface of tube to the bottom half. Thus, the fluid at the bottom section could also be warm. The latter generates the secondary flow if it would be enough temperature differences. Since the warmer fluid tends to move upward and the colder goes down. In the case of higher Richardson number, the secondary flow is well established and significantly affects the fluid flow. Hot flow from the near wall region goes up and then backs downward at the centreline region. While at the lower *Ri*, where the circumferential temperature variation is low the strength of secondary flow is low. By tube inclination the warmer fluid is more accumulated at the upper part of tube because of decreasing secondary flow strength. Increasing the tube inclination augments the near wall axial buoyancy force while the radial component decreases. Thus, the strongest secondary flow vector is seen in the case of horizontal tube.

**Figure 6:**
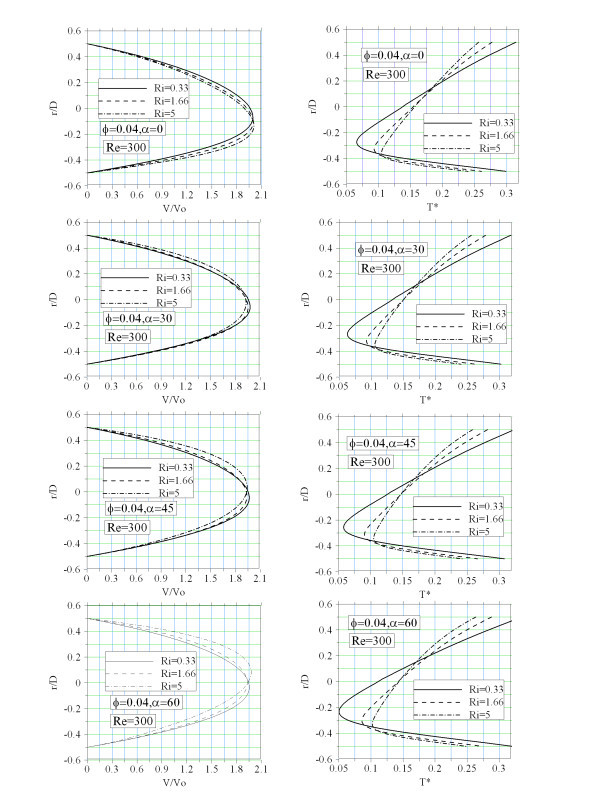
**Fully developed axial velocity and temperature profile**.

To see how the axial velocity profile is affected by the secondary flows, Figure 6 is presented. This figure shows the effect of tube inclination and the Richardson number on the axial velocity profile and dimensionless temperature profile. At *α *= 0, increasing the Richardson number shifts the position of maximum axial velocity toward the bottom section. Since, the strength of secondary flow augments with the Richardson number. As intended, by increasing the Richardson number the bottom half of tube is also more affected by the energy that is transferred from the top half of tube. The temperature variation at the tube cross-section augments. By increasing the tube inclination temperature variation at the tube cross-section becomes more uniform. The latter tends to shift the maximum axial velocity towards the upper part of tube. Where, axial component of the buoyancy forces is more important.

**Figure 7:**
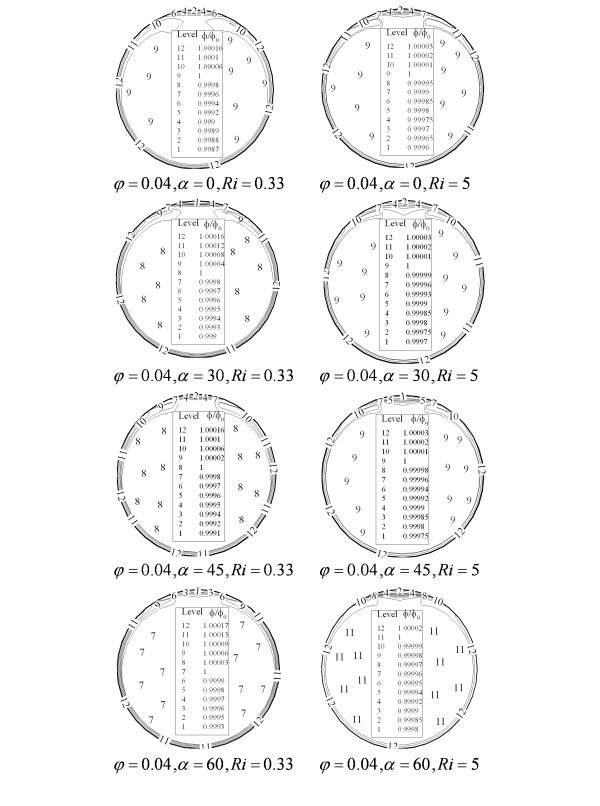
**Nanoparticles distribution at different Ri and tube inclinations**.

**Figure 8:**
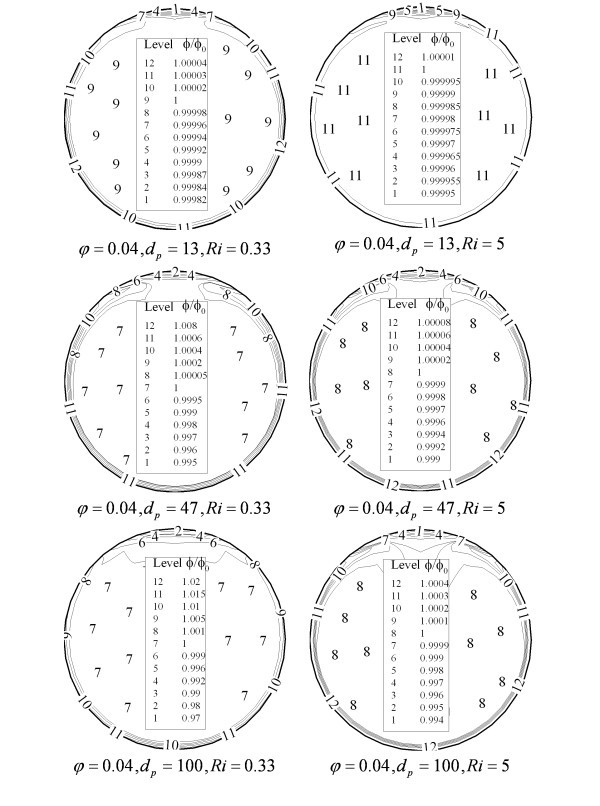
**Effect of nanoparticle mean diameter on the homogeneity of nanofluid**.

As seen in Figures 7 and 8, these forces and the secondary flow induced by the cross-sectional component of the buoyancy forces affects the homogeneity of the dispersed nanoparticles. At the near wall region where the effect of viscous layer is more significant, nanoparticle concentration is more evident. In the other hand, secondary flow causes to see a region of lower nanoparticle concentration at the top of tube where the direction of circular cell changes and goes back toward the bottom of tube. Thus, higher tube inclinations improve the homogeneity of the nanoparticles distribution. As shown in Figure 8 using larger particle accelerates the migration of the nanoparticle and deteriorates the nanofluid homogeneity. For the particles with smaller mean diameter, this variation is not significant and thus homogeneous distribution could be considered. While increasing nanoparticle mean diameter, non-uniformity on the particles distribution becomes more important and single-phase approach may fail. These effects could significantly affect heat transfer throughout the tube.

**Figure 9:**
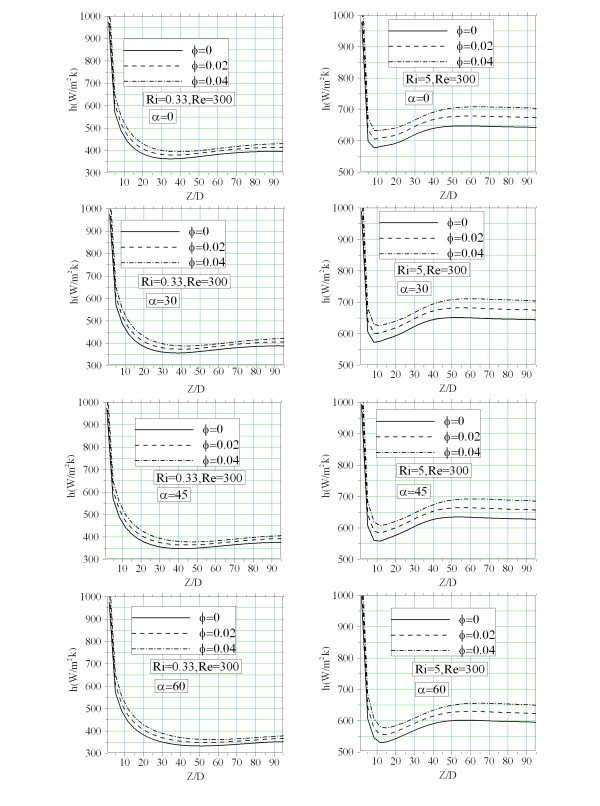
**Axial evolution of the peripheral average convective heat transfer coefficient**.

Axial evolution of the average peripheral convective heat transfer coefficient along the tube length is shown in Figure 9. In general *h *decreases and monotonically goes to its asymptotic value. Buoyancy forces components (axial and radial) significantly affected the variations of heat transfer coefficient. At the lower *Ri *for which the effect of buoyancy force is weak, maximum heat transfer coefficient could be seen in the case of horizontal tube (pure radial buoyancy force). While at the higher Richardson number the buoyancy forces augments and so both axial and radial components become considerable. Based on the value of the axial and radial components of the buoyancy force, the best tube inclination for which the highest heat transfer coefficient is achieved could be determined. For instance, at the low *Ri*, horizontal configuration gives the best heat transfer coefficient (among the other angle in Figure 9) while for the higher Richardson number (*Ri *= 5) it appears at tube inclination of *α *= 30. This behaviour is also seen for different nanofluids. However, using nanofluid enhances heat transfer coefficient. This enhancement becomes more important at the higher Richardson number.

**Figure 10:**
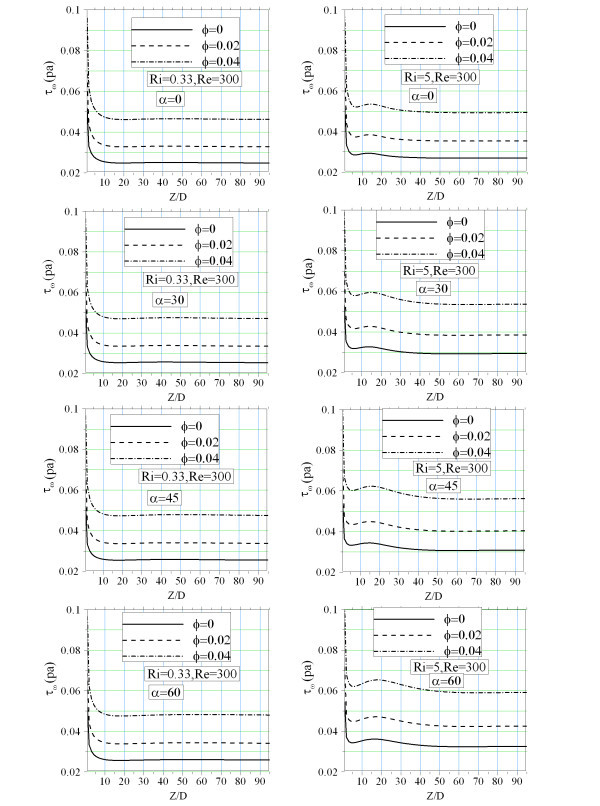
**Axial evolution of the peripheral average wall shear stress**.

In spite of increasing heat transfer coefficient the peripheral average shear stress is also augmented. This is shown in Figure 10 for different tube inclination and nanoparticle volume fraction. As seen, increasing nanoparticle concentration augments the shear stress which means more pumping power is needed for the fluid pumping. This partially arises from the fact that nanofluid viscosity increases with the nanoparticle concentration. The axial buoyancy forces and near wall fluid acceleration has an important effect on the shear stresses. Thus, by increasing the tube inclinations axial buoyancy forces augments and the higher value of the average shear stress is observed. This is more evident in the case of higher Richardson number. To have a comparison between the heat transfer enhancement and pressure drop augmentation with nanoparticle concentration detail analysis of the corresponding data in the case of *Re *= 300 and *Ri *= 5, is presented as an example. It shows that increasing the nanoparticle volume fraction from 0 to 2%, pressure drop augments by about 31% while the heat transfer coefficient increases by about 5%. This is showed that despite of stability and homogeneity of the nanofluids the pumping power is also an important concern that must be well addressed.

## Conclusion

Conjugate laminar mixed convection of water/Al_2_O_3 _nanofluid in an inclined copper tube has been investigated numerically by using two-phase mixture model. The top half of tube wall is heated while the other half of tube is considered to be adiabatic. Copper is a good conductive material and transfers the heating energy from the upper part of tube to the lower half of tube by heat conduction mechanism. This could also increase the fluid temperature at this region. The latter could generate the secondary flow for which its strength depends on the nanoparticle volume fraction, the Richardson number and tube inclination angle. The buoyancy induced secondary flow augments with the nanoparticle volume fraction and the Richardson number. However, by tube inclination the axial component of the buoyancy forces increases and so the strength of secondary flow decreases. Nanoparticle concentration does not have significant effect on the axial velocity profile. However, at the high value of the Richardson number for which the effect of thermal energy is become more important than the hydrodynamic energy, nanoparticle concentration could affect the axial velocity profiles. Heat transfer coefficient is augmented with the nanoparticle volume fraction as well as the Richardson number. Combinations of the axial and radial component of the buoyancy forces could determine the inclination angle for which the maximum heat transfer enhancement occurs. However, the wall shear stress is significantly increased with the nanoparticle volume fraction. It is also augmented with the tube inclination because of increasing the axial component of the buoyancy forces.

## Abbreviations

**List of symbols**: C_p_: Specific heat; *D*: Tube diameter (m); *d_f_*: Molecular diameter of base fluid; *d*_p_: Nanoparticle diameter (nm); *E*: Energy (J/kg); *gd*_f_: Gravitational acceleration (ms^-1 ^); *Gr*: Grashof number ; *h*: convection heat transfer coefficient ; *k*: Thermal conductivity (W/m K); *P*: Pressure (Pa); *Pr *Prandtl number ; q": Average heat flux at the solid-fluid interface (W/m^2^); *r*: Radial direction (m); r_i_: Tube radial inner (m); r_o_: Tube radial outer (m); *Re*: Reynolds number ; Ri: Richardson number ; *T*: Temperature (K); T*: Temperature dimensionless ; *t*: Thicknesses (m); *V*: Velocity (m/s); *Z*: Axial direction. 

**Greek letters**: α: Tube inclination; β: Volumetric expansion coefficient (K^-1^); θ: Angular coordinate; ϕ: Volume fraction; μ: Dynamic viscosity; ν: Kinematic viscosity; ρ: Density; τ: Shear stress. 

**Subscripts**: b: Bulk; dr: Drift; eff: Effective; o: Outer condition; p: Particle; f: Base fluid; i: Inner condition; k: Summation index; m: Mixture; nf: Nanofluid; s: Solid; w: Interface; 0: Inlet condition.

## Competing interests

The authors declare that they have no competing interests.

## Authors' contributions

SA carried out the numerical simulation as a part of his master thesis which has been done under supervision of AB. SMS was adviser of the thesis. The authors have had regular scientific meeting and discussions during the work. All Authors read and approved the final manuscript.
